# Early detection of hypovolemia by venous pulse wave velocity and vena cava ultrasound imaging

**DOI:** 10.1007/s00421-025-06050-3

**Published:** 2025-11-12

**Authors:** Marco Romanelli, Leonardo Ermini, Piero Policastro, Ruben Allois, Luca Mesin, Paolo Pasquero, Silvestro Roatta

**Affiliations:** 1 Integrative Physiology Lab, Department of Neuroscience, Universitá di Torino, Turin, Italy; 2 Mathematical Biology and Physiology, Department of Electronics and Telecommunications, Politecnico di Torino, Turin, Italy; 3 Department of Medical Sciences, Universitá di Torino, Turin, Italy

**Keywords:** Pulse wave velocity, Lower body negative pressure, Inferior vena cava, Collapsibility index, Volemic status

## Abstract

**Purpose:**

Non-invasive assessment of the volemic status is still an open and urgent clinical need. The pulse wave velocity in the venous compartment (vPWV) has recently been re-proposed as a possible indicator but still needs to be tested in different settings including hypovolemic conditions.

**Methods:**

The present study aims at investigating the response of the vPWV to hypovolemic changes of different magnitude, as provided by the lower body negative pressure (LBNP) and using the ultrasound monitoring of the inferior vena cava (IVC) as a reference. In 28 healthy subjects, vPWV was measured at the arm during exposure to short (90 s at -10, -20, -30, -40 mmHg) and long (5 min at -30 mmHg) exposure to LBNP, along with arterial blood pressure (ABP) and heart rate (HR). In a subgroup of 17 subjects IVC was successfully monitored and the full-time course of IVC diameter (dIVC) and collapsibility indices were extracted from the recording.

**Results:**

While collapsibility indices were little responsive, vPWV and dIVC were significantly affected by LBNP, decreasing by 19.5 ± 9.7 and 20.9 ± 21.3% at -10 mmHg, respectively. Only the respiratory caval index exhibited significant increases at LBNP ≤ 30mmHg. As compared to other variables vPWV exhibited best reproducibilty: coefficient of variation < 5%.

**Conclusion:**

vPWV appears to be a promising non-invasive indicator of blood volume decrease with early high sensitivity and reproducibilty.

## Introduction

Today, the assessment of volume status remains an open issue and a crucial aspect in fields such as fluid management, dialysis, and heart failure (Androne et al. 2004; Dekker and Kooman 2018; Argaiz et al. 2021). In particular it is often necessary to detect early signs of increase or decrease in blood volume, for instance when monitoring fluid responsiveness and rehydration in hypovolemic patients (Gelman and Bigatello 2018; Monnet and Teboul 2018) or the effects of ongoing volume loss by, e.g., diuretics, dialysis or hemorrhage (Pacagnella et al. 2013; Pasquero et al. 2015; Argaiz et al. 2021). Since the majority (approximately 70%) of circulating blood is contained in the venous compartment (Monnet and Teboul 2018), this is the primary target of different assessment methodologies.

The direct measurement of central venous pressure (CVP), via a catheter inserted in a central vein has been discouraged, due to the risks associated with the invasive procedure as well as to the poor clinical usefulness (Marik et al. 2008), in favour of non-invasive approaches, mostly based on ultrasound (US) (Monnet and Teboul 2020; Kearney et al. 2022). Ultrasound monitoring of large central veins, such as the inferior vena cava (IVC) (Nagdev et al. 2010; Bortolotti et al. 2018), or the jugular vein (Uthoff et al. 2012; Pellicori et al. 2014; Rizkallah et al. 2014), as well as Doppler US of abdominal veins (Argaiz 2021), are non-invasive and widely used methods for assessing volume status and venous congestion. Two methodologies are here considered and briefly introduced: the classical US imaging of the IVC and the emerging venous pulse wave velocity (vPWV).

In recent years, a variety of image processing algorithms have been developed to address the major limitations of US-based assessment of the IVC (Policastro et al. 2023), so as to improve its otherwise poor reliability (Orso et al., 2020). In particular, automatic IVC tracking allowed to reduce movement artifacts introduced by the respiratory displacement of the IVC (Mesin et al. 2015), averaging the IVC size over an entire longitudinal segment reduced the variability associated with the arbitrary choice of a specific point (Mesin et al. 2019b); selectively filtering the time course of the IVC diameter made it possible to appreciate the relevance of the cardiac oscillatory component (Nakamura et al. 2013) in the global IVC collapsibility and to separately quantify a cardiac caval index (CCI) and a respiratory caval index (RCI) (Mesin et al. 2020; Ermini et al. 2022). In particular, it was shown that (1) RCI and CCI may be uncorrelated, suggesting that they carry different information (Ermini et al. 2022; Policastro et al. 2025) and (2) it is possible to perform long-lasting recordings to follow the time course of these hemodynamic indices in response to different manoeuvres. However, one unaddressed limitation of US IVC monitoring is the subject- and context- dependent poor imaging quality, possible caused by the subcutaneous adipose tissue layer and the intestinal gas. Moreover, the responsiveness of the collapsibility index, often also termed “caval index” (CI), to hypovolemic stimuli is debated (Resnick et al. 2011; Pasquero et al. 2015; Bussmann et al. 2018; Johnson et al. 2021).

An alternative methodology for objective assessment of volume status was recently reconsidered. Original studies in the seventies proposed that the vPWV could be a sensitive indicator of blood volume changes (Felix et al. 1971; Nippa et al. 1971). However, the idea was not followed up, possibly due to technical difficulties and high variability of the measurement. The introduction of technical improvements in the methodology recently demonstrated the feasibility of the measurement, with different approaches (Ermini et al. 2020; George et al. 2024). It was shown that vPWV can detect increases in venous pressure in the lower limbs (femoral vein) in response to passive trunk elevation (Ermini et al. 2020) and increases in blood volume in upper limbs (basilic vein) in response to passive leg raising (PLR) (Ermini et al. 2021), while vPWV responsiveness to blood volume depletion has never been investigated in humans.

The present study aimed to investigate the responsiveness of vPWV to hypovolemic stimuli of different magnitude and duration in comparison to the indications obtained from simultaneous US imaging of IVC, including the IVC diameter, the CI, and its respiratory and cardiac components RCI and CCI, respectively. In particular, the different variables were characterized in terms of sensitivity and reproducibility, and the concordance was assessed between vPWV and IVC-derived parameters. In addition, possible sex differences will also be investigated as a stronger vasoconstrictive response to LBNP In males than females was previously observed and a lower tolerance to hypovolemic/orthostatic stress is often reported for females, compared to males (Franke et al. 2003; Fu et al. 2004; Yang et al. 2012; Carter et al. 2015). Hypovolemic stimuli were conveniently delivered through exposure to lower-body negative pressure (LBNP), a method commonly used in both research and clinical settings to study orthostatic tolerance and cardiovascular responses to reversible hypovolemic challenges (Goswami et al. 2019).

Additional measurements, including near-infrared spectroscopy (NIRS) monitoring of the upper and lower limbs, were also conducted as part of this experimental series and have been addressed in a separate study (Romanelli et al. 2025).

## Materials and methods

### Subjects

Twenty-eight subjects were enrolled: 16 males (age: 25±9 years, weight: 75± 9 kg, height: 177± 6.1 cm; BMI: 23.8 ± 3.0 Kg·m^2^), and 12 females (age: 23± 3 years, weight: 57± 8 kg, height: 164± 3.4 cm; BMI: 21.2 ± 3.2 kg·m^2^). The US IVC imaging was successful recorded only in a subgroup of 17 subjects (IVC-subgroup: 9 M / 8 F, age: 21± 2 / 22± 3 years, weight: 70± 6.4 / 57± 8 kg, height: 176± 3.6 / 164± 3.5 cm; BMI: 22.5± 2.5 / 21.1 ± 3.3 kg·m^2^). Participants were excluded from the study if presenting prior history of cardiovascular disease or susceptibility to hypotensive crises and fainting episodes. The study was conducted in agreement with the principles of the Declaration of Helsinki (2000) and under the approval of the Ethics Committee of the University of Torino (n. 0059551, 30-1-2023). All subjects gave their written informed consent.

### Experimental set-up

**Fig. 1:**
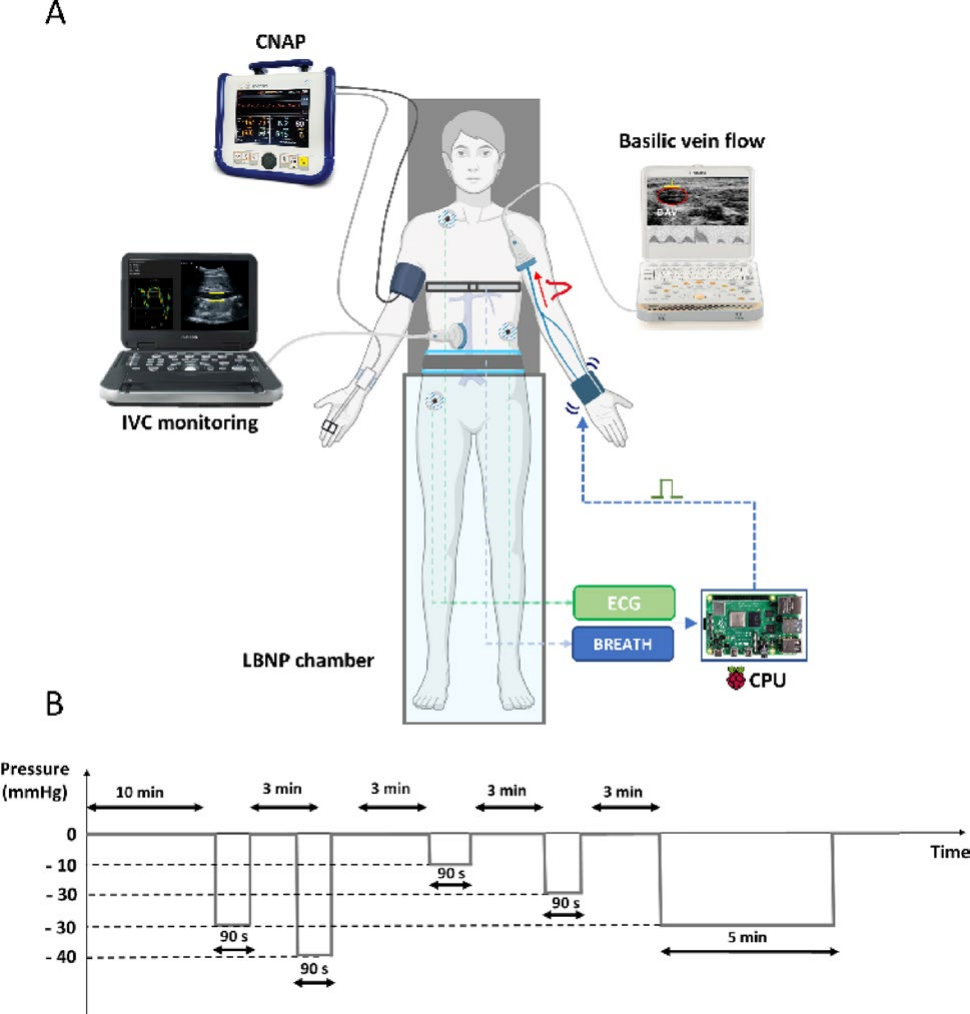
Experimental setup (**A**), including the arterial blood pressure monitor (CNAP), two ultrasound devices to monitor blood flow in the basilic vein and size changes in the IVC, the control unit (CPU), which triggers cuff inflations at the wrist, based on respiratory and cardiac activity, and the LBNP chamber. Experimental protocol (**B**): the sequence of the 90 s lasting stimuli was randomized, while the 5 min lasting stimulus always followed at the end

A scheme of the experimental set-up is provided in Fig. 1A. Arterial blood pressure (ABP) was continuously and non-invasively monitored by finger photo-plethysmography (CNAP system, CNSystems Medizintechnik, Graz, Austria), along with cardiac output (CO), mean arterial pressure (MAP), heart rate (HR), and total peripheral resistance (TPR). This device was placed on the subject’s right hand, while the left arm was concerned by the vPWV measurement as previously described (Ermini et al. 2021). In short, a venous pressure pulse periodically generated at the wrist and propagating proximally is detected close to the axillary cavity and the propagation velocity is computed. The pressure pulse is generated by a pneumatic cuff (GIMA pediatric cuff, 7.5 × 26 cm) wrapped around the left wrist rapidly inflated by compressed air (inflation time: 50 - 60 ms, peak pressure: 400–500 mmHg, duration: 100-150 ms, interval between subsequent compressions: about 15 s). The wrist compressions are delivered synchronously with the heartbeat, during the end-expiratory phase to eliminate the influence of cardiac and respiratory activity on the venous blood flow. To this aim an electrocardiographic and a respiratory band signal need to be acquired by the control unit (see below). The pressure pulse is detected proximally, at the level of the basilic vein, by a Doppler-US (MyLab 25 Gold, ESAOTE, Genova, Italy) through a linear probe (12.5 MHz, model LA523), oriented transversally at an angle of about 60 deg to the vessel long axis, and maintained in the same position for the entire duration of the experiment by a probe holder. The cuff-probe distance is set as large as possible to minimize the relative error in its measurement, performed manually (Boutouyrie et al. 2008). The Doppler audio signal is also acquired and processed by the control unit, consisting of single board computer (Raspberry Pi), wirelessly connected to a PC, where a graphic interface allows for interacting with the device. This unit thus implements all the necessary task for the vPWV measurements: (1) triggering the delivery of the pneumatic compression at the wrist, based on ECG and respiratory signals; (2) acquiring the Doppler signal in a subsequent 0.5-s time window; (3) processing the Doppler signal to detect the footprint of the propagated pulse; (4) calculating the vPWV as the ratio among the travelled distance (Δx) from the cuff to the US probe and the propagation time, also known as pulse transit time (PTT), equal to the time interval between the cuff inflation and the footprint in the Doppler signal; (5) saving and displaying the calculated vPWV values. More details have been provided in a previous report (Barbagini et al. 2022).

The upper part of the IVC was visualized using a subxiphoid approach with two-dimensional (B-mode) longitudinal US imaging (Ecube-i7, ALPINION, Rome, Italy), equipped with a convex 2–5 MHz probe. Images were obtained with the patient in the supine position during relaxed, normal breathing (Mesin et al. 2020; Ermini et al. 2022, p. 202). Continuous video output of the US system was supplied to a PC by means of a frame grabber (Vinmooog HDMI Video Capture; Shenzhen Yiminmin Trading Co., Ltd., Shenzhen, China) and recorded with Spike2 Video Capture tool by CED (Cambridge Electronic Design) (frame rate 30 Hz, 1920 × 1080 pixels).

As mentioned above, NIRS measurements from resting forearm and thigh muscles were also recorded but are not discussed in the present study.

Hypovolemic stimuli were provided by a LBNP chamber (LBNP 1100, TECHNAVANCE, Texas, USA). Magnitude and duration of the negative pressure stimuli were programmed in advance through its digital interface (described below). All signals were digitally sampled (1401micro acquisition board and Spike2 software, CED, Cambridge, UK, ) at a sampling frequency of 100 Hz, except for the echo Doppler signal that was acquired with a sampling rate of 6250 Hz, and continuously recorded for the whole experimental session, for offline analysis (MATLAB^®^ R2023b, The MathWorks Inc., Natrick, Massachusetts, United States).

### Experimental protocol

Participants were instrumented and positioned supine within the LBNP chamber sealed at the level of the iliac crest. The distance between the cuff, placed on the wrist, and the US probe, placed near the axillary cavity, was measured and typed into the vPWV device. Each experimental session started with the subject in supine position resting for at least 10 min, to allow for equilibration of fluid compartments (Montgomery et al. 1977). Measurement of vPWV through the periodic delivery of wrist compressions and US IVC monitoring were continuously performed throughout. The protocol consisted of four randomized LBNP stimuli (-10, -20, -30, -40 mmHg, 90-s duration), followed by a final long-lasting stimulus (-30 mmHg, 5-min duration), all separated by 3-min resting intervals (Fig. 1B). The isolated short-lasting stimuli were meant to investigate the effect of hypovolemic stimuli of different magnitude while avoiding the cumulative effect produced by longer lasting, contiguous stimuli. The longer lasting exposure to -30 mmHg was aimed to investigate the occurrence of the possible slower changes in venous blood volume. The entire experimental protocol lasted about 35 min.

### Data processing

**Fig. 2:**
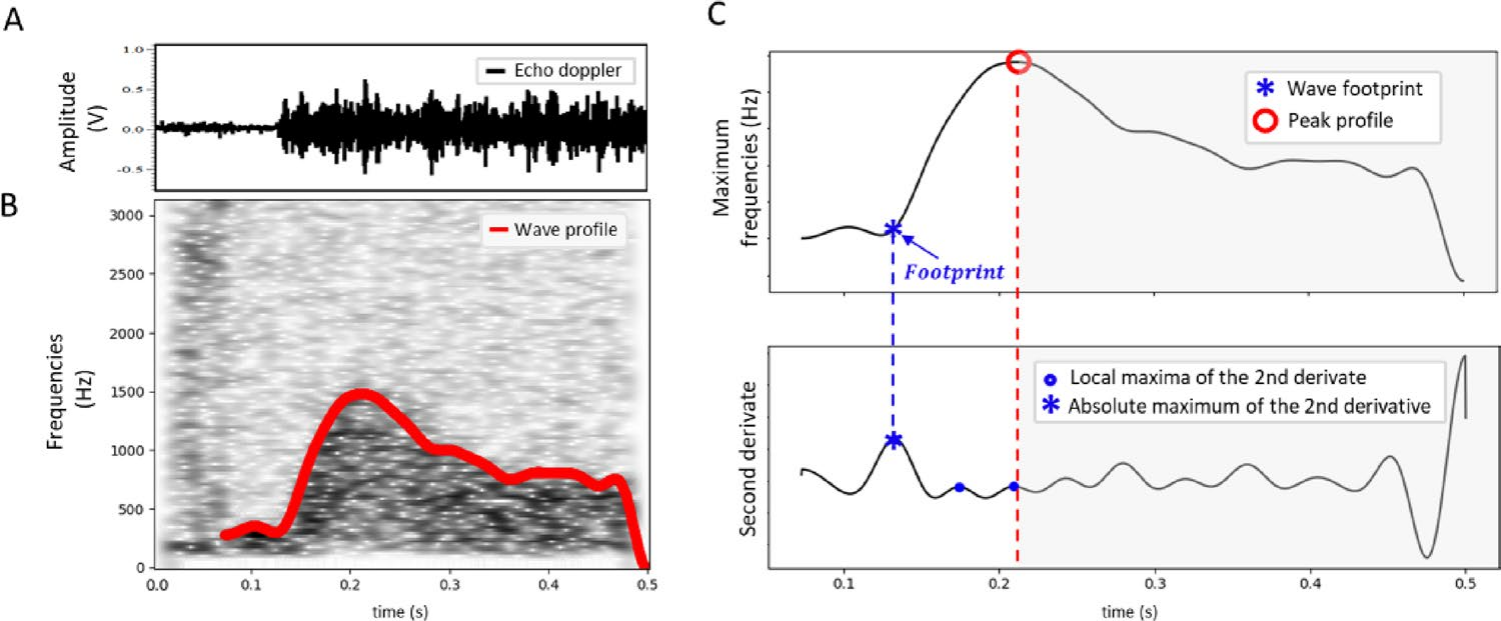
Processing of a single Doppler signal epoch for the measurement of the Pulse Transit Time (PTT): from the echo Doppler signal (**A**), the maximum blood velocity profile was extracted by using the spectrogram (**B**). The wave footprint was then identified as the maximum peak of the second derivative of this velocity profile (**C**)

The calculation of vPWV includes the following steps (Fig. 2): acquisition of a 500 ms epoch of the echo-Doppler signal collected from the basilic vein, starting from the instant of cuff inflation; identification of the wave footprint; calculation of PTT as the time distance from cuff inflation to the footprint; calculation of vPWV as the cuff-to-Doppler-probe distance divided by PTT. A time-frequency domain analysis was performed on the echo-Doppler signal (Figure 2A) using the spectrogram, from which the maximum frequency profile (equivalent to maximum blood velocity or outline) was extracted (Figure 2B). The footprint of this profile was identified as the highest local maximum of its second derivative (Chiu et al. 1991; Boutouyrie et al. 2008), occurring before the time of peak velocity (red dashed line in Fig. 2C). All the signal processing steps were performed in Python^®^.

**Fig. 3:**
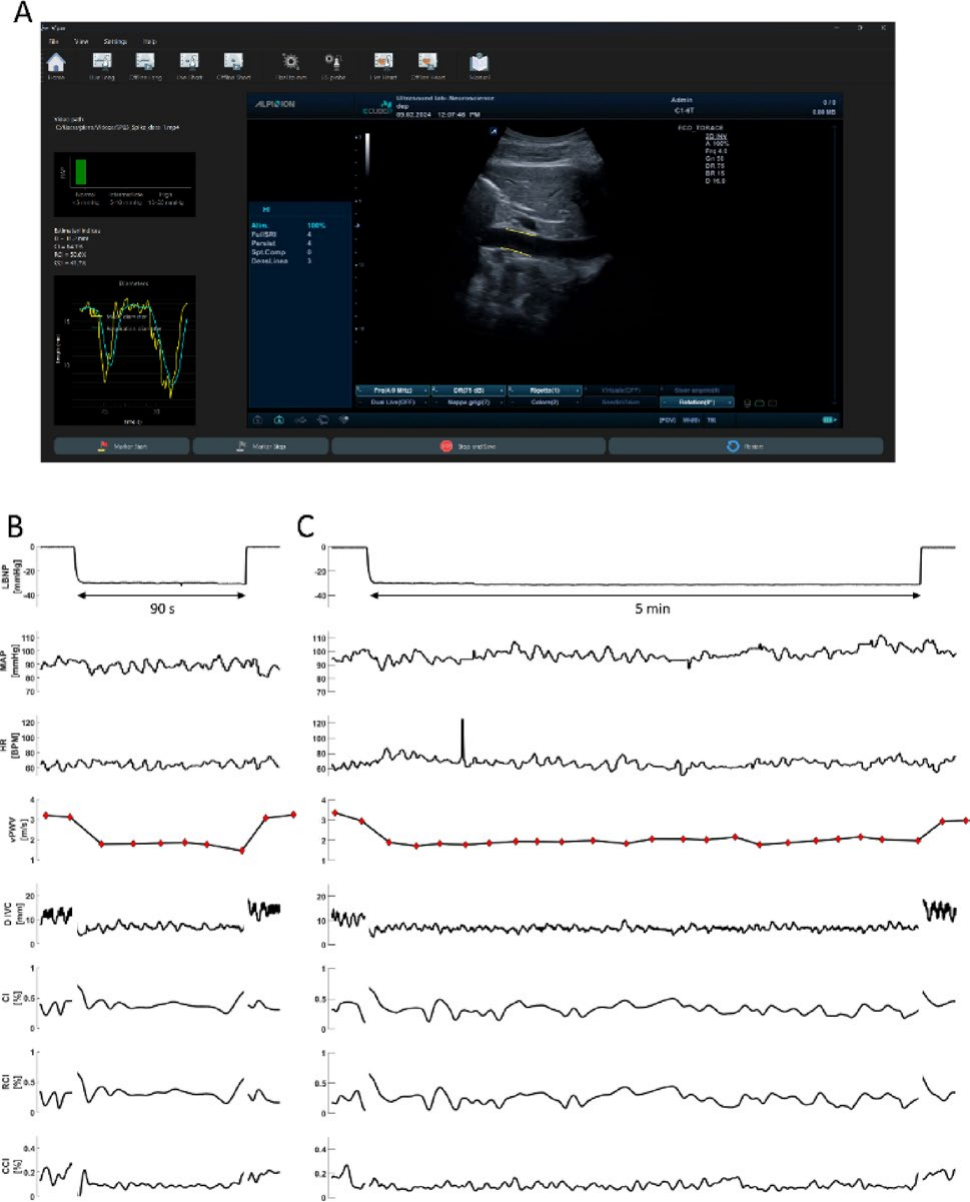
Graphical user interface for off-line processing of the video clip, showing the segmentation of the longitudinal section of the IVC (highlighted in yellow) and the calculation of the different indices (**A**). Time course of the parameters recorded during LBNP at -30 mmHg lasting 90-s (**B**) and 5-min (**C**), from a representative subject. The start and end of LBNP are marked with vertical red lines. From top to bottom: pressure in the LBNP chamber (LBNP), mean arterial pressure (MAP), heart rate (HR), venous pulse wave velocity (vPWV), inferior vena cava diameter (D IVC), caval index (CI), respiratory caval index (RCI), cardiac caval index (CCI). The processing of IVC imaging was interrupted for about 1 s at the onset and at the end of the LBNP stimulus to exclude movement artifacts

IVC segmentation in US videos was conducted using a bespoke software package, specifically designed for IVC edge-tracking (Viper, Torino, Italy) (Figure 3A). The routines employed were based on a previously established algorithm (Mesin et al. 2019b) able to estimate, frame by frame, the IVC diameter (dIVC), the CI, the RCI and the CCI (Mesin et al. 2020; Ermini et al. 2022). In short, the CI is classically defined as the difference between the maximum (Dmax) and minimum IVC diameter, divided by Dmax over a respiratory cycle. As for RCI and CCI, they are obtained from the same formula after isolating the respiratory and the cardiac oscillatory components, respectively, by band-pass filtering. Enhancements were made to the tracking algorithm to mitigate the effects of minor drifts, which could adversely impact the long-duration videos considered in this study (Policastro and Mesin 2023; Policastro et al. 2024).

For each hypovolemic stimulus, two intervals were identified, corresponding to the 20 s before the beginning of the stimulus (baseline) and to the last 30 s before the end, i.e. at 90 s (T_90s_), or at 5 min (T_5min_) and time averages were calculated. For the all parameters, a unique average baseline value was obtained from the 4 baselines pertaining to the four 90-s stimuli. To quantify the further changes taking place when extending the duration of the stimulus from 90 s to 5 min, the average values collected at T_90s_ and at T_5min_ of the 5-min lasting stimulus were compared to baseline. Average values of CI, RCI and CCI were computed after performing a Fisher Z-transformation as required for indices ranging between 0 and 1 (Ermini et al. 2022). Finally, two reproducibilty tests were conducted on vPWV, dIVC, CI, RCI and CCI: the first one by comparing the values obtained from each of the four 20-s periods preceding each LBNP stimulus and the second one by comparing the measurements collected at T_90s_ from the two 30-mmHg stimuli (of 90-s and 5-min duration).

The whole signal processing was performed in MATLAB (MATLAB (R2023b), Natick, Massachusetts: The MathWorks Inc.).

### Statistical analysis

The statistical analysis was done using IBM^®^ SPSS^®^ Statistics 28.0 (IBM corp., Armonk, New York, United States). The normality of the data was checked by the Shapiro-Wilk’s test, and homoscedasticity by the Levene’s test.

Subjects’ age, weight, height, BMI, and baseline values of the CNAP variables (ABP, HR, CO and TPR) and vPWV in the 90-s tests were compared between males and females, using a two-tailed unpaired sample t-test. In the IVC sub-group, the same analysis was repeated, with the additional parameters derived from the IVC assessment (dIVC, CI, RCI, CCI).

A two-way mixed Analysis of variance (ANOVA) was performed to investigate the effects of LBNP (0, -10, -20, -30 and -40 mmHg) and sex (male and female) on CNAP variables and vPWV over all subjects. The test was repeated over the IVC sub-group and extended to IVC variables. In case of significant main effects, a Least Significant Difference (LSD) post-hoc test was performed to analyze specific differences. In addition, a two-way mixed ANOVA was performed to calculate the effects of time (baseline, T_90s_, and T_5min_) and sex over all subjects and over the IVC sub-group, in response to the 5-min, -30 mmHg exposure.

The sensitivity, reproducibility and concordance of vPWV and IVC variables (dIVC, CI, RCI CCI) were assessed in the IVC sub-group. The sensitivity was calculated for vPWV and dIVC in response to the weakest stimulus, as the ratio between the percentage change in the variable and the magnitude of the stimulus (10 mmHg). The reproducibility of the measurement was assessed both under baseline conditions, using the Coefficient of Variation (CoV) calculated as standard deviation /mean·100, and at T_90s_ of -30mmHg exposures using Pearson’s R^2^ correlation coefficient. The concordance between vPWV and dIVC was individually calculated by assessing the Pearson’s *r* correlation coefficient among the five numerical pairs of values collected at 0, -10, -20, -30, -40 mmHg and then averaged over all subjects. The concordance between vPWV and collapsibility indices was similarly computed, but a Fischer’s transform was applied to individual *r* values before averaging and the anti-transform was then applied to the obtained average.

The results are presented as mean ± standard deviation; p-values less than 0.05 were considered significant.

## Results

As anticipated in the Methods section, US monitoring of the IVC in long axis of sufficiently good quality could be obtained only in 17 (9 M, 8 F) out of 28 subjects, due to poor echogenicity of some of the subjects. An example of satisfactory US image quality, with automatic identification of the IVC is reported in Fig. 3A. Average anthropometric and physiological parameters are distinctly presented in Table 1 for males and females in resting supine position and with reference to the whole subject group and the IVC subgroup. Although sharing the same age, males presented with higher weight, height and BMI and lower HR (BMI and HR, not reaching significance in the IVC sub- group), while no differences were detected on the other variables.

**Table 1:** Male vs. Female comparisons in the all-subjects group and the IVC subgroup, in resting conditions

Parameter	All subjects			IVC sub-group		
	Males (*n*=16)	Females (*n*=12)	Significance	Males (*n*=8)	Females (*n*=7)	Significance
Age	24.5±9.5	22.8±2.8	*p*=.60	21.8±2.0	22.4±3.0	*p*=.42
Weight (kg)	74.6±9.2	57.0±7.8	*p*<.05	63.8±6.4	56.9±8.4	*p*<.05
Height (cm)	177.1±6.1	164.3±3.4	*p*<.05	176.3±3.6	164.4±3.5	*p*<.05
BMI (kg/m^2^)	23.8±3.0	21.2±3.2	*p*<.05	22.5±2.5	21.1±3.3	*p*=.338
MAP (mmHg)	87.10±7.3	86.9±10.1	*p*=.94	85.15±5.9	88.0±12.2	*p*=.54
HR (BPM)	63.3±10.9	73.1±12	*p*=.09	64.8±6.8	72.7±14.9	*p*=.18
CO (l/min)	6.17±1.3	6.3±1.1	*p*=.54	6.06±1.3	6.3±1.3	*p*=.41
TPR (mmHg/l/min)	13.73±3.1	13.6±2.9	*p*=.88	13.61±2.2	13.7±3.5	*p*=.97
vPWV (m/s)	3.17±0.6	2.8±0.4	*p*=.11	3.19±0.5	2.9±0.5	*p*=.25
dIVC (mm)	/	/	/	11.96±4.9	11.6±5.4	*p*=.88
CI (%)	/	/	/	0.47±0.2	0.30±0.1	*p*=.15
RCI (%)	/	/	/	0.30±0.2	0.20±0.1	*p*=.13
CCI (%)	/	/	/	0.24±0.1	0.20±0.1	*p*=.22

*BMI* body mass index, *MAP* mean arterial pressure, *HR* heart rate, *CO* cardiac output, *TPR* total peripheral resistance, *vPWV* venous pulse wave velocity, *dIVC* inferior vena cava diameter, *CI* caval index, *RCI* respiratory Caval index, *CCI* cardiac caval index

### Exposure to 90-s LBNP

Exposure to 90-s LBNP, ranging from -10 to -40 mmHg, was well tolerated by all subjects. The entire time course of the different variables and of the calculated indices, from a representative subject exposed to -30 mmHg, is shown in Fig. 3B. It can be observed that, while HR and ABP were almost unaffected by the LBNP, vPWV decreased sharply by approximately 50% at LBNP onset, then remained relatively stable throughout the stimulus. The dIVC exhibited a qualitatively similar pattern of reduction, often transiently achieving complete collapse in the very first phase of the response. Note that in the lower traces the full-time course of the collapsibility indices is reported, none of them exhibiting a clear response to LBNP. All variables promptly return to control values at the end of the stimulus.

**Fig. 4:**
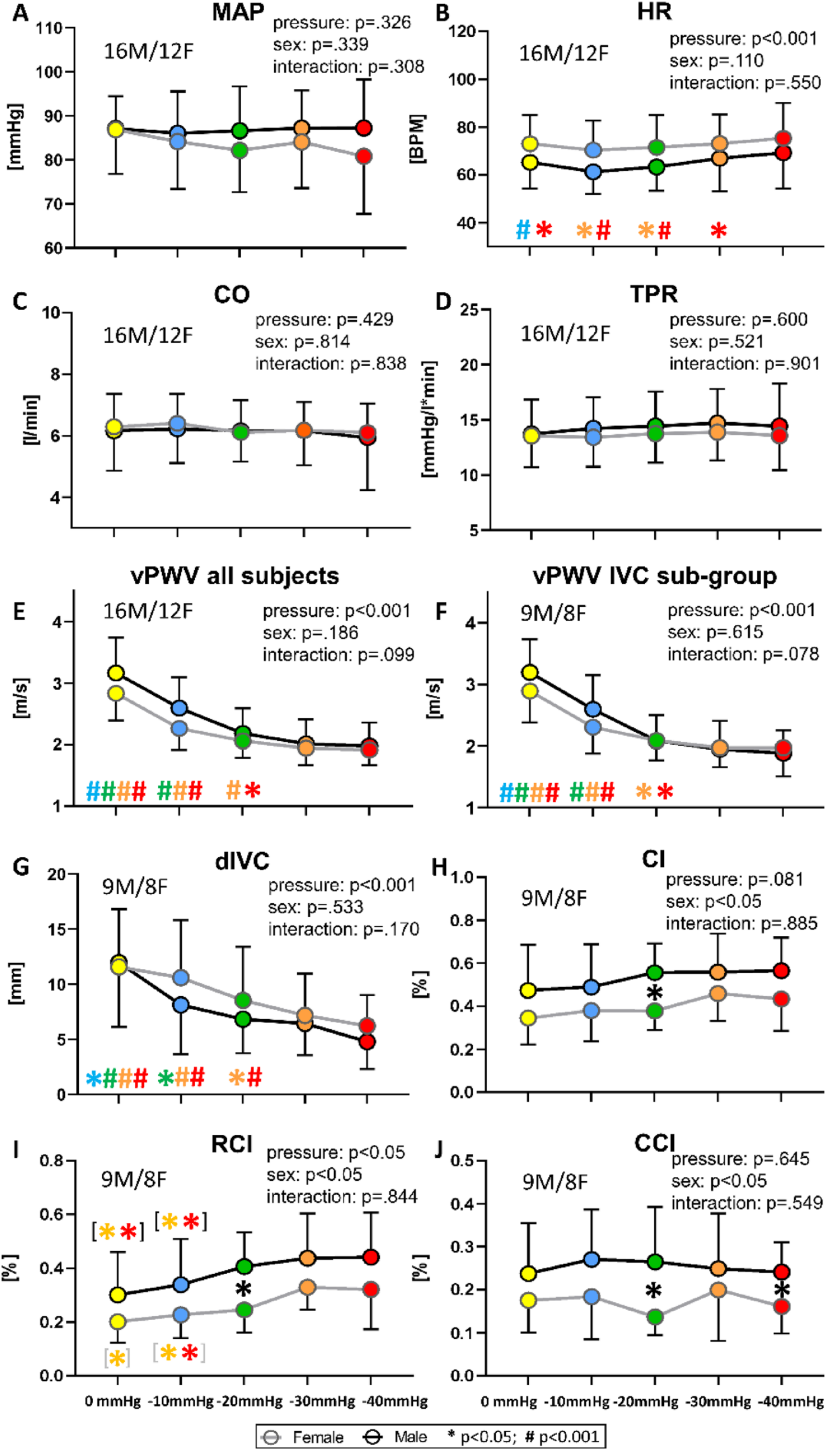
Average of LBNP on systemic variables (**A–D**), vPWV (**E**,** F**) and IVC indices (**G–J**) in males and females. From top to bottom: mean arterial pressure (MAP), heart rate (HR), cardiac output (CO), total peripheral resistance (TPR), venous pulse wave velocity (vPWV), inferior vena cava diameter (dIVC), caval index (CI), respiratory caval index (RCI), cardiac caval index (CCI). Different colours are associated with the different pressure levels, while the black and grey lines indicate males and females, respectively. Main effects and interaction are reported in text form and post hoc tests are reported with symbols (#: *p* < .001; * *p* < .05), the colour identifying the pressure level considered in the comparison, e.g., a red asterisk over the green dot indicates a significant difference between the red (40 mmHg) and green (20 mmHg) conditions. If # and * are within black (gray) square brackets they refer specifically to the male (female) group. Otherwise, they refer to the whole group (males + females). The total number of subjects (n) is also distinctly specified for males (M) and females (F)

A comparison of the responses between males and females to the various magnitudes of LBNP, along with statistical significance, is presented in Fig. 4. No significant changes from baseline were observed, nor were there any sex differences in MAP, although females tended to show a slight decrease with increasing LBNP intensity. As previously mentioned, a sex difference was observed in HR, with females showing higher values than males, and significant dependence on pressure but no sex-pressure interaction. No main effects were instead detected in CO and TPR. Both vPWV and dIVC significantly decreased at different levels of negative pressure, with no dependence on sex. Note that the vPWV vs. the LBNP curve remains unchanged when calculated over the IVC-subgroup only. Finally, CI and RCI exhibited a slight upward trend with increasing the LBNP, only RCI achieving statistical significance, while CCI was unaffected by LBNP. A sex difference was observed in all three collapsibility indices, post-hoc analysis revealing this difference at -20 mmHg and, in CCI only, at -40 mmHg.

### Exposure to 5-min LBNP

Exposure to -30 mmHg for 5-minute was well tolerated by all subjects. The time course of the hemodynamic responses of a representative subject, is shown in Fig. 3C.

It can be observed that even this longer lasting exposure did not produce major effects on ABP and HR all effects previously described for the 90-s exposure are essentially maintained for the whole 5-min duration, although a slow recovery trend is visible in the vPWV tracing.

**Fig. 5:**
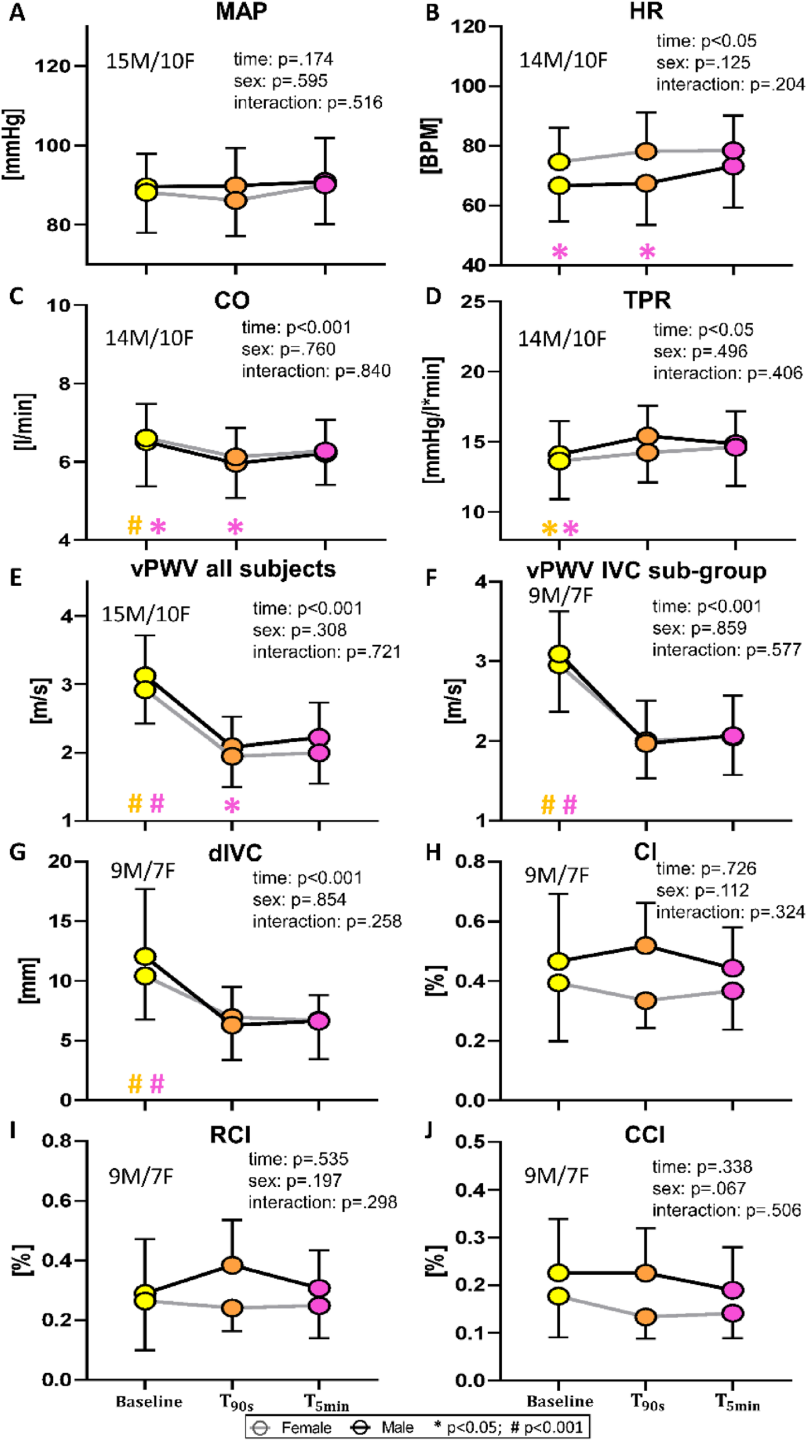
Comparison of average changes at 90 s (T90s) and 5 min (T5min) from the beginning of a −30 mmHg LBNP stimulus. The diagrams show changes in systemic variables (**A–D**), vPWV (**E**, **F**) and IVC indices (**G–J**) as shown in Figure 4. Different colours are associated with the different times, while the black and grey lines indicate males and females, respectively. Main effects and interaction are reported in text form and post hoc tests are reported with symbols (#: *p* < .001; * *p* < .05), the colour identifying the time instant considered in the comparison, as reported in Fig. 4

Averaging over all subjects allowed to evidence that small but significant changes took place between 90 s and 5 min, namely, a slight increase in HR, CO, dIVC and vPWV, as shown in Fig. 5. No difference was observed on IVC indices and on vPWV when calculated over the IVC subgroup.

### Sensitivity, reproducibility and concordance

Comparison of vPWV and IVC variables, limited to the IVC subgroup, was quantitatively assessed as reported in Table 2. The sensitivity was similar between vPWV and dIVC in response to -10 mmHg LBNP exposure (IVC indices were not significantly affected). The reproducibility of the different variables was first assessed over the 4 baselines intervals preceding the 90-s tests. It was observed that vPWV exhibited the lowest CoV (4.6±2.7%), as compared to CI, RCI and CCI. In addition, the reproducibility was assessed by correlating the two measurements collected in hypovolemic conditions (-30 mmHg, at T_90_): similar Pearson’s R² values were observed. Finally, the vPWV exhibited a good concordance with dIVC (*r* = .89) and progressively worse with RCI, CI and CCI.

**Table 2:** Sensitivity, reproducibility and concordance between the indices related to the two approaches used to evaluate changes in blood volume

Indices	Sensitivity	Reproducibility		Concordance
	(%/mmHg)	CoV (%) (between the baselines)	Pearson’s *r*^2^ (at -30mmHg)	Pearson’s *r* (vPWV vs. others)
vPWV IVC sub-group	-2.6±1.6	4.6±2.7	0.69	~
dIVC	-2.1±2.1	11.0±9.6	0.69	0.89
CI	n.s.	18.3±11.7	0.70	-0.39
RCI	n.s.	26.7±13.9	0.71	-0.58
CCI	n.s.	20.8±12.3	0.53	0.12

*CoV* Coefficient of variation, *vPWV* venous pulse wave velocity, *IVC* inferior vena cava, *dIVC* inferior vena cava diameter, Caval index (CI), *RCI* respiratory caval index, *CCI* cardiac caval index

## Discussion

### Summary of results

For the first time, vPWV was investigated in response to hypovolemic stimuli, obtained by short-lasting isolated applications of LBNP in the range -10 to -40 mmHg, and the results were compared with US imaging of IVC. The main findings are summarized as follows:

1. vPWV could be effectively monitored throughout the experimental protocol in all subjects, without facing the limitation encountered by abdominal US (see below).
2. vPWV exhibited good reproducibilty and high sensitivity to the early stage of hypovolemia, decreasing by 19.6% at the lightest LBNP level of -10 mmHg (26% in the IVC subgroup).
3. US IVC imaging was successful in only about half of the subjects due to poor echogenicity. The IVC size decreased with LBNP while the CI and CCI did not show the expected increasing trend, exhibited only by RCI.
4. Females exhibited lower values of IVC collapsibility indices than males at baseline and in hypovolemic conditions.

### vPWV, a marker of venous filling

In a previous study, it was shown that vPWV can detected large increases in venous pressure, as obtained in the legs during progressive trunk elevation from a supine position (Ermini et al. 2020). Subsequently, it was shown that vPWV can also detect more subtle changes in blood volume unaccompanied by major changes in venous pressure, as obtained in the arms in response to passive leg raising (Ermini et al. 2021). Whether vPWV is also sensitive to blood volume depletion was only investigated in early studies, mostly conducted on animal models and with a more rudimental methodology (Yates 1969; Felix et al. 1971). In particular, it was shown that vPWV, measured in the abdominal vena cava (Yates 1969) and in the superior vena cava (Felix et al. 1971) of dogs exposed to haemorrhage, decreased according to the reduction in venous pressure. It was also noted that vPWV exhibited early and greater changes than arterial blood pressure and heart rate, in response to the blood loss (Felix et al. 1971).

In the present human study, a blood loss was simulated by the LBNP which, by producing a blood shift towards the legs, lowers venous pressure in the upper body (Hinojosa-Laborde et al. 2014; Johnson et al. 2014).

Exposure to LBNP ranging between -10 and -20 mmHg is generally considered a mild stimulus, equivalent to a blood loss of 400-550 ml (~ 10% of the total blood volume) while stimuli between -20 and -40 mmHg are considered of moderate intensity and equivalent to a 500-1000 ml blood loss (Cooke et al. 2004; Goswami et al. 2019). Based on a computational human model of haemorrhage, Summers et al., estimated a blood displacement to lower limbs of 486, 664 and 938 ml to result from LBNP of -15 -30 and -60 mmHg, respectively, in a 70-kg subject (Summers et al. 2009).

Attempts to compare LBNP exposure to haemorrhage in terms of changes in CVP were performed by Hinojosa-Labrode in primates reporting LBNP of -22±6 mmHg to match a blood loss of 6.25% and a LBNP of -41±7mmHg for a 12.5% blood loss (Hinojosa-Laborde et al. 2014), with CVP being decreased to about 2 and 0.5 mmHg in the two conditions, respectively. These values match those reported for clinically relevant conditions such as following a 2-kg fluid loss during dialysis (Thalhammer et al. 2015) or experimental dehydration, by a 20-min sauna at 80 °C (Nakamura et al. 2016). As compared to other techniques for inducing hypovolemic/hypotensive stimuli such as the head-up tilt), the LBNP has the important advantage of maintaining the subject in the supine position, without introducing hydrostatic pressure gradients among different body regions. Consequently, the specific effects of venous pressure changes can be investigated, including those on the vessel wall stiffness and PWV, as described by the Bramwell-Hill equation (Bramwell and Hill 1922).

The present study evidenced a sharp decrease in vPWV (-19.6%), already at the lightest LBNP of -10 mmHg, with a response curve that appears to saturate at -30/40 mmHg. Although the literature reports quite variable decreases of CVP by LBNP in healthy subjects, ranging from 1 to 3.4 mmHg at -10 mmHg of LBNP (Pawelczyk and Raven 1989; Duranteau et al. 1991; Rea et al. 1991; Peters et al. 2000; Furlan et al. 2001), by assuming a CVP decrease of 2.36 mmHg, equal to the average of the reported effects, an initial sensitivity of about 0.24 m/s/mmHg can be calculated. Interestingly, a much lower sensitivity of about 0.023 m/s/mmHg characterized the response to increases in venous pressure, as observed in a previous study in which venous pressure in the legs was raised by progressive trunk elevation from the supine position (Ermini et al. 2020).

**Fig. 6:**
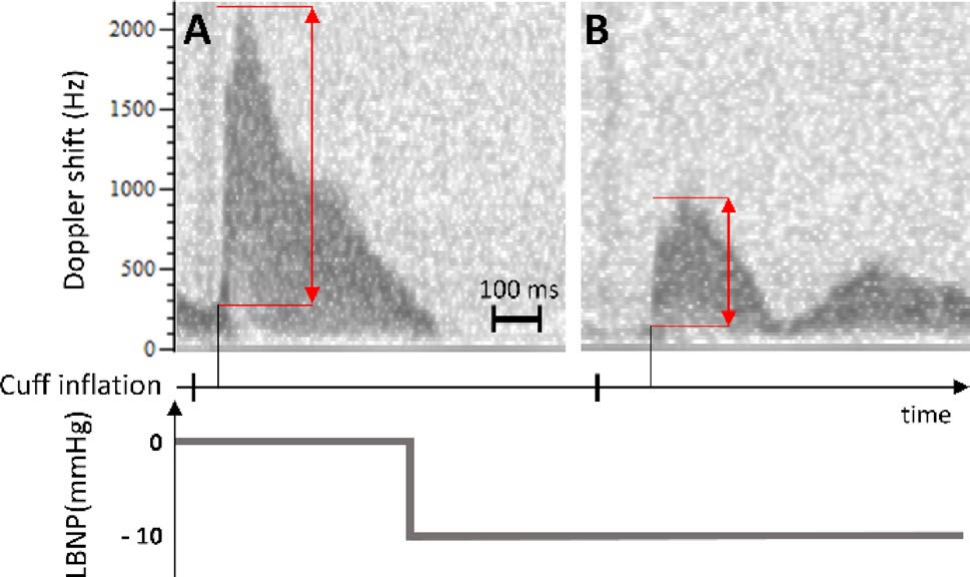
Blood velocity curves from the same subject at baseline (**A**) and during the hypovolemic stimulus at -10 mmHg (**B**). The trigger lines at the bottom indicate the time instant of the cuff inflation, the red arrows indicate the wave amplitude, while the black lines are placed in correspondence with the footprint. Note the lower magnitude of the pulse wave and increased latency from the cuff inflation indicating reduction in vPWV during the LBNP (**B**). The time scale is reported in the lower right corner of (**A**)

In spite of the many approximations, like assuming similar changes in central and peripheral venous pressures, and of the experimental differences (vPWV measured in arms or legs) the high sensitivity of vPWV to the initial stage of hypovolemia may be explained by two different factors. First of all, the non-linear volume-pressure relation of venous vessels reveals much higher steepness (i.e., compliance) at lower pressures (Halliwill et al. 1999; Mesin et al. 2022). Secondly, the PWV is also affected by the magnitude of the pulse wave: a phenomenon that is often neglected but that was demonstrated in a recent investigation, specifically for the vPWV (Ermini and Roatta 2023). In particular, with the same set-up adopted in the present study, it was shown that pressure waves of lower magnitude, generated by weaker pneumatic compressions of the wrist, propagated to the basilic vein at a lower velocity (Ermini and Roatta 2023). Incidentally, it was here observed that pneumatic compressions of unchanged magnitude produced weaker pulse waves, as detected by Doppler US at the basilic vein (see the representative recording in Fig. 6), during LBNP compared to baseline. This effect is likely due to the decreased efficacy of the pneumatic compression when LBNP partially empties tissues and venous vessels from blood, as documented by the decrease of blood volume indices from NIRS monitoring of the upper arm during LBNP stimuli (Romanelli et al. 2025), but could also be related to increased dampening of pressure waves in hypovolemic conditions (Alian et al. 2014).

In conclusion, by virtue of the cumulative effects of the above mechanisms, the vPWV presents as a sensitive marker of hypovolemia.

### IVC monitoring

The dIVC confirmed to be a good and early indicator of blood volume reduction, exhibiting significant changes already at -10 mmHg and progressively decreasing up to -30/-40 mmHg. The data are in agreement with previous studies based on LBNP (Moore et al. 2010; Johnson et al. 2021) as well as other models of hypovolemia: Johnson et al. (2021) showed a significant decrease of maximum and minimum dIVC at -20 mmHg of LBNP, and similar results were reported in blood donors after a 400-450-ml blood subtraction (Lyon et al. 2005; Resnick et al. 2011; Pasquero et al. 2015). However, small changes in IVC diameter may have poor clinical significance given the large interindividual variability in IVC size (Resnick et al. 2011), although the issue is debated (Clemency et al. 2022). In this respect, the CI should offer a more robust indication of vascular filling, being virtually independent of IVC size. However, it does not provide a clear-cut univocal response to real or simulated decreases in blood volume. In response to blood donation, both a significant CI increase from 35 to 45% in long axis IVC monitoring (Pasquero et al. 2015) and no significant changes (Resnick et al. 2011) were reported. Changes of different sign with no significant net effect were reported in response to venesection (Bussmann et al. 2018). Even in response to LBNP up to -40 mmHg the CI did not exhibit a significant increase in a group of young healthy men (Johnson et al. 2021). Also, the present results report just a trend of increase of CI with LBNP magnitude, not achieving statistical significance, thus confirming this general picture. However, they also add new insights and possibly help to understand the poor CI responsiveness. The IVC oscillatory pattern is generally considered to be of respiratory origin with only a minor “disturbance” from cardiac activity. However, recent investigations by appropriate filtering, managed to separate the cardiac from the respiratory component showing that the former provides a non-negligible contribution to the whole oscillation (Nakamura et al. 2013; Sonoo et al. 2015; Mesin et al. 2019b, 2020). This can be readily appreciated also from the present data, by observing the reduction of collapsibility index when the cardiac component is filtered out (i.e. compare CI with RCI values in Figs. 4 and 5). It can then be observed that only RCI, and not CCI, significantly increases with LBNP and we can only speculate about the possible reasons. It is possible that decreased heart filling and decreased CVP have contributed to the dampening of the back-propagating cardiac pressure wave, thus blunting the increase in CCI expected from the increased IVC compliance during LBNP. This interpretation is supported by the study of Alian et al. (Alian et al. 2014) reporting strong attenuation (> 60%) of the cardiac modulation of peripheral venous pressure in the upper arm during LBNP at -30 mmHg. Furthermore, the magnitude of cardiac pulse waves in peripheral venous pressure, as assessed through the spectral power of the relevant frequency band, was shown to decrease with blood donation, hemorrhage and diuretics (Miles et al. 2018; Alvis et al. 2020). Along the same line, the transmission of the right atrial pressure to abdominal veins increases with increasing venous congestion (Argaiz 2021).

Conversely, the respiratory component of venous pressure appears to be unaffected by hypovolemia (Alian et al. 2014). Therefore, the significant increase in RCI observed in the present study during LBNP may reflect the increased IVC compliance. The possibility that it could be caused by an increase in depth of respiration is excluded, based on previous studies (Ahn et al. 1989; Convertino et al. 2009).

In conclusion, the present results, emphasizes the importance of separating cardiac and respiratory components in IVC collapsibility, as they are differently affected by different factors. They also suggest that the CCI, that was proven to effectively detect increases in cardiac preload (Nakamura et al. 2013; Ermini et al. 2022), may not as well perform in response to hypovolemic challenges.

### vPWV vs. IVC monitoring

Both vPWV and dIVC appear to be early marker of hypovolemia with comparable sensitivity (Table 2) to low-magnitude stimuli, with comparable dependence on LBNP (concordance of 0.89). As the LBNP magnitude increases, they both exhibit a *floor* effect, with stimuli at -40 mmHg producing no further significant decreases in vPWV and dIVC, as compared to -30 mmHg. A likely explanation for the floor effect is that CVP itself is reported to approach the lowest limit of 0 mmHg at LBNP of 30-40 mmHg (Peters et al. 2000; Furlan et al. 2001; Hinojosa-Laborde et al. 2014; Rickards et al. 2015). In fact, although slightly negative values have also been reported (Johnson et al. 2014), a negative venous pressure is prevented outside the thoracic cavity by venous collapse, thus resulting in a floor effect.

The low CoV, which characterizes the vPWV measurement in resting conditions (4.6%), confirms the results of previous reports and the appropriateness of the improvements implemented in the methodology (Ermini et al. 2021; Barbagini et al. 2022), namely, the synchronization of the pulse wave generation with cardiac and respiratory activities, which reduces the disturbance originating from their modulation of venous blood flow and pressure. In comparison, the CoV of dIVC and IVC collapsibility indices settles at much higher levels (above 10%), as observed also in previous reports (Mesin et al. 2019a; Ermini et al. 2022), although the reproducibilty assessed in hypovolemic conditions (T90 at -30 mmHg) was similar among the different parameters (R^2^ around 70% in correlating the two measurements).

A further aspect to be considered is the feasibility of the measurement. Good quality ultrasound IVC monitoring is challenged by several causes such as adipose tissue and gas in the bowel. Moreover, due to the variability of spontaneous breathing, continuous tracking of IVC size and averaging over long time intervals is required to obtain reliable estimates of dIVC and collapsibility indices. On the other hand, vPWV requires a delicate set-up, which includes cardiac and respiratory monitoring, application of a peripheral pneumatic cuff, and continuous ultrasound monitoring of blood flow in the basilic vein. This latter measurement requires that the arm of the subject is maintained still for the duration of the recording or that the probe position is continuously controlled and adjusted to maintain the quality of the Doppler recording. In addition, an excessive pressure exerted by the US probe on the skin may partially occlude the vein, possibly affecting the upstream venous pressure and the vPWV measurement. These constraints currently prevent an easy translation to the clinical setting. Adoption of possible alternative techniques to detect the venous pulse wave (e.g., tonometry, bioimpedance), currently under investigation, may improve the feasibility of the measurement. An alternative methodology to assess the vPWV of spontaneous waves in the jugular vein has also been proposed. Improved methodologies may facilitate the investigation of vPWV in patient populations.

### Sex differences

To date, no studies in the literature have specifically investigated sex-related differences in vPWV. The present data do no evidence significant differences in either resting or LBNP conditions. Additionally, males and females exhibited similar dIVC values both at baseline and during hypovolemia, in agreement with the findings of Franco et al., stating that dIVC differences typically appear from the age of 51 onwards (Franco et al. 2022).

Surprisingly, males exhibited higher IVC collapsibility indices than females, also in basal conditions. The issue of sex differences in IVC pulsatility appears to be little explored. Few studies, carried out in a children population reported absence of sex differences (Taneja et al. 2018; Ghosh et al. 2021; Mathews et al. 2023), while no reference was found with relevance to the adult population.

It seems unlikely that this difference really indicates an increased IVC compliance in females, possibly associated with lower vascular filling, since dIVC was similar in the two groups and data from the literature report no differences in CVP basal conditions (Lalande et al. 2015). Besides, anatomical differences may partly explain the effect. Women, generally have a smaller rib cage, a diaphragm approximately 9% shorter, and a greater rib inclination than men and, for these reasons, tend to rely more on thoracic respiration than men (Bellemare et al. 2003; LoMauro and Aliverti 2018). However, this has implications in IVC collapsibility, since during abdominal and not thoracic inspiration abdominal pressure increases, thus exerting a compressive action on the IVC (Macklem 1981; Folino et al. 2017; Mesin et al. 2022) and increasing its collapsibility, as also shown experimentally during diaphragmatic and thoracic breathing (Kimura et al. 2011; Byeon et al. 2012).

While this reasoning may explain the lower CI and RCI in females than in males, a different mechanism should be responsible for the lower CCI. One possibility is that a lower pulse wave is generated by the females ’heart, considering that it is also characterized by a smaller size and lower stroke volume (Franke et al. 2003; St. Pierre et al. 2022), which would then result in lower cardiac IVC pulsatility. Based on the above speculative considerations, the reported sex-differences are unlikely to have clinical relevance.

### Limitations

In this study we compared the performance of vPWV to IVC-based indicators of vascular filling. Admittedly, the ultrasound IVC monitoring is affected by many limitations and a direct comparison with a gold standard, would have been more appropriated for validating the vPWV methodology. The invasive assessment of CVP is often adopted to this purpose (Hinojosa-Laborde et al. 2014; Johnson et al. 2021; Mastrandrea et al. 2024). However, in recent years many authors have discouraged the use of CVP as an indicator of volemic status and fluid responsiveness, in favor of dynamic indices derived from US-based techniques and stroke volume and CO changes in response to fluid challenges (Michard and Teboul 2002; Marik et al. 2008; Monnet and Teboul 2020; Kearney et al. 2022)., In addition to its possible validation in heathy subjects, future studies on patient populations are necessary to assess the actual usefulness of vPWV in monitoring the patient’s hemodynamic status and in the management of the fluid therapy.

The analysis of IVC size has been performed with an automated software which tracks possible vessel displacements and automatically detects the vessel wall, for calculation of vessel size. While this provides many advantages including, continuous monitoring over large time intervals of vessel size and collapsibility, objectivity, minimization of movement artifacts, and noise reduction thanks to averaging the measurement over a whole longitudinal IVC segment, it requires medium-good quality US imaging. This may have caused the exclusion of some of the subjects presenting with poor echogenicity, thus further increasing the dropout of subjects from the IVC group. In addition, the automated diameter measurement may not drop precisely to 0 when IVC collapses either because the collapse does not extend over the whole length of the analysed segment or because of the thickness of vessel walls which is not completely accounted for. This may have occasionally led to slight overestimation of the minimum IVC diameter and underestimation of its collapsibility.

## Conclusions

For the first time, vPWV, a newly reconsidered index of blood volume, has been tested in response to hypovolemic challenges of different magnitude. The results evidence that vPWV outperforms the classical collapsibility index of the IVC in terms of sensitivity to reductions in central blood volume, a reproducibilty of the measurement and a success rate of the assessment in healthy subjects, while providing similar sensitivity and good agreement with dIVC. Even though the methodological constraints of the present implementation present a relevant limitation to its usability, the results encourage future applications and validations in the clinical setting.

## Data Availability

Data will be made available upon reasonable request.

## Abbreviations

ABP: Arterial blood pressure
ANOVA: Analysis of variance
CCI: Cardiac caval index
CI: Caval index
CO: Cardiac output
CoV: Coefficient of variation
CVP: Central venous pressure
Dmax: Maximum diameter
dIVC: IVC diameter
HR: Heart rate
IVC: Inferior vena cava
LBNP: Lower-body negative pressure
MAP: Mean arterial pressure
NIRS: Near-infrared spectroscopy
PTT: Pulse transit time
RCI: Respiratory caval index
TPR: Total peripheral resistance
US: Ultrasound
vPWV: Venous pulse wave velocity
