# Correction: Early detection of hypovolemia by venous pulse wave velocity and vena cava ultrasound imaging

**DOI:** 10.1007/s00421-025-06119-z

**Published:** 2026-03-17

**Authors:** Marco Romanelli, Leonardo Ermini, Piero Policastro, Ruben Allois, Luca Mesin, Paolo Pasquero, Silvestro Roatta

**Affiliations:** 1 Integrative Physiology Lab, Department of Neuroscience, Universitá di Torino, Turin, Italy; 2 Mathematical Biology and Physiology, Department of Electronics and Telecommunications, Politecnico di Torino, Turin, Italy; 3 Department of Medical and Sciences, Universitá di Torino, Turin, Italy

**Correction: European Journal of Applied Physiology (2025)** 10.1007/s00421-025-06050-3

In the original version of this article, the given name and family names of authors were incorrectly structured. The correct given name and family name should be

Marco Romanelli

Leonardo Ermini

Piero Policastro

Ruben Allois

Luca Mesin

Paolo Pasquero

Silvestro Roatta

Affiliation 3 details were incorrectly given as ‘Department of Medical and Specialist Sciences, Universitá di Torino, Turin, Italy’ but should have been ‘Department of Medical and Sciences, Universitá di Torino, Turin, Italy’.

The original article has been corrected.

